# Incontinentia Pigmenti: Learning Disabilities Are a Fundamental Hallmark of the Disease

**DOI:** 10.1371/journal.pone.0087771

**Published:** 2014-01-29

**Authors:** Maria Rosa Pizzamiglio, Laura Piccardi, Filippo Bianchini, Loredana Canzano, Liana Palermo, Francesca Fusco, Giovanni D'Antuono, Chiara Gelmini, Livia Garavelli, Matilde Valeria Ursini

**Affiliations:** 1 IRCCS, Santa Lucia Foundation, Rome, Italy; 2 Life, Health and Environmental Science Department, University of L'Aquila, L'Aquila, Italy; 3 Psychology Department, Sapienza University, Rome, Italy; 4 Institute of Genetics and Biophysics “Adriano Buzzati Traverso”, Naples, Italy; 5 Clinical Genetics Unit, Obstetrics and Pediatric Department, Istituto di Ricovero e Cura a Carattere Scientifico, Arcispedale Santa Maria Nuova Hospital, Reggio Emilia, Italy; 6 Fondazione IRCCS SDN, Naples, Italy; Alexander Fleming Biomedical Sciences Research Center, Greece

## Abstract

Studies suggest that genetic factors are associated with the etiology of learning disabilities. Incontinentia Pigmenti (IP, OMIM#308300), which is caused by mutations of the IKBKG/NEMO gene, is a rare X-linked genomic disorder (1∶10000/20∶000) that affects the neuroectodermal tissues. It always affects the skin and sometimes the hair, teeth, nails, eyes and central nervous system (CNS). Data from IP patients demonstrate the heterogeneity of the clinical phenotype; about 30% have CNS manifestations. This extreme variability suggests that IP patients might also have learning disabilities. However, no studies in the literature have evaluated the cognitive profile of IP patients. In fact, the learning disability may go unnoticed in general neurological analyses, which focus on major disabling manifestations of the CNS. Here, we investigated the neuropsychological outcomes of a selected group of IP-patients by focusing on learning disabilities. We enrolled 10 women with IP (7 without mental retardation and 3 with mild to severe mental retardation) whose clinical diagnosis had been confirmed by the presence of a recurrent deletion in the IKBKG/NEMO gene. The participants were recruited from the Italian patients' association (I.P.A.SS.I. Onlus). They were submitted to a cognitive assessment that included the Wechsler Adult Intelligence scale and a battery of tests examining reading, arithmetic and writing skills. We found that 7 patients had deficits in calculation/arithmetic reasoning and reading but not writing skills; the remaining 3 had severe to mild intellectual disabilities. Results of this comprehensive evaluation of the molecular and psychoneurological aspects of IP make it possible to place “learning disabilities” among the CNS manifestations of the disease and suggest that the IKBKG/NEMO gene is a genetic determinant of this CNS defect. Our findings indicate the importance of an appropriate psychoneurological evaluation of IP patients, which includes early assessment of learning abilities, to prevent the onset of this deficit.

## Introduction

Incontinentia Pigmenti (IP, OMIM#308300) is a rare X-linked dominant genomic disorder that is lethal in males. Females can survive thanks to X-inactivation mosaicism [1], [2]. The gene responsible for IP, which is called the Inhibitor of Kappa light polypeptide gene enhancer in B-cells, Kinase Gamma/Nuclear factor kappaB Essential MOdulator (*IKBKG/NEMO,* NM_003639.3; OMIM#300248) [3] is located in Xq28 and encodes for a regulatory subunit of NF-kappaB signalling, which is involved in many physiological functions [4], [5], [6]. Most IP patients have an identical deletion (*IKBKGdel*) that eliminates the genomic region from exon4 to exon10 of the *IKBKG/NEMO* gene and consequently abolishes the protein function [2], [7], [8].

The phenotypic expression of IP is always characterized by skin lesions. They appear at birth and evolve spontaneously in four typical inflammatory stages, which are currently considered as the diagnostic criteria for IP [1], [9]. Frequently, other neuroectodermal tissues including teeth, hair, nails, eyes and the central nervous system (CNS) are also damaged [2], [10], [11]. Moreover, a marked variability of the phenotype is also present in related patients with the same genetic alteration [2]. The CNS is affected in 10–30% of IP cases, resulting in seizures, hemiparesis, spasticity, microcephaly, cerebellar ataxia and mental retardation [2], [10], [11]. This inconsistent involvement of the CNS in IP is a typical aspect of the highly heterogeneous clinical presentation always reported in this disease [2], [10], [11]. Indeed, abundant data have shown that the same *IKBKG/NEMO* mutation (*IKBKGdel*) might produce intrafamilial heterogeneity with mild IP in the mother and severe IP in the daughter [2]. It has been assumed that this wide range of variability is due to the random selection of X-inactivation in heterozygous IP females, coupled with the pleiotropic role of the NEMO/IKKgamma protein in the cell. In fact, the NEMO/IKKgamma is involved in a complex signalling pathway that regulates the expression of hundreds of genes, and its mutation can produce different, unpredictable phenotypic outcomes. Indeed, this explains the wide spectrum of anomalies observed in IP.

Neuroimaging investigations of IP patients have shown infarctions (often multiple) and atrophy [12], [13]. In addition to white matter abnormalities, both large-vessel and small-vessel diseases have been reported [13]. Some studies have suggested the presence of pathogenic mechanisms, including inflammatory, developmental, vascular and infectious processes. Hennel *and colleagues* [13] suggested that small-vessel occlusion is the primary cause of this condition and the inflammatory process is a secondary cause. However, the IP-induced pathogenesis of the CNS is still a controversial issue. It has been suggested that developmental inflammatory mechanisms, occlusive phenomena in small vessels, or both, are responsible [10], [11]. In almost all IP patients with CNS lesions, clinical neurological abnormalities, such as seizures, mental delay and hemiparesis, have been found [11], [14]. In particular, studies have reported that one-third of all IP patients are affected by seizures and mental retardation [15] as a consequence of neurological damage [16]. Moreover, the absence of structural CNS abnormalities does not exclude the possibility that brain functions are altered. To our knowledge the cognitive phenotype of this syndrome has never been investigated using standard neuropsychological instruments that allow describing the cognitive profile of IP. The NEMO/IKKgamma protein is, however, involved in a complex signalling pathway that regulates the expression of hundreds of genes and its mutation can produce different phenotypic outcomes.

In any case, studying the cognitive phenotype of IP in detail should provide new insights for a better understanding of this illness.

The presence of specific *learning disabilities* is likely overlooked in IP patients who have no neurological or intellectual abnormalities, because until now attention has been focused on the predominant manifestations that have the greatest impact on quality of life. Learning disabilities include a group of disorders characterized by inadequate development of specific academic, language, and speech skills. For example, learning disabilities affect reading, mathematics and writing. We focused on the investigation of learning disabilities because more than 150 genes on the X-chromosome have been identified as responsible for learning disabilities [17]; in fact, they account for over 10% of all cases of learning disabilities [18]. As the IP syndrome is an X-linked dominant genomic disorder, we hypothesized that some as yet un-assessed learning disabilities might be present in patients without CNS signs symptoms. Furthermore, it is worth of noting that NEMO/IKBKG is the crucial regulator of the entire NF-kB transcription pathway, which has been shown to be required for synaptic plasticity and learning in mice [19], [20] . Therefore, we aim to perform a detailed evaluation of the cognitive phenotype of IP in order to further improve our knowledge about this illness.

Using a multidisciplinary approach, we aimed to investigate the cognitive phenotype of a selected group of IP patients to determine whether any CNS dysfunctions cause significant difficulty in the acquisition and use of listening, speaking, writing, reasoning or mathematical abilities. Moreover, as IP is diagnosed at birth, IP children with *learning disabilities* could benefit from early and appropriate therapeutic support to prevent onset of the cognitive deficit.

## Methods

### Participants

Between February and December 2012, we enrolled ten women with Incontinentia Pigmenti (IP) who had a defined *IKBKG*/*NEMO* gene deletion (*IKBKGdel*) in Xq28. Participants were recruited through the Italian patients' association (I.P.A.SS.I. Onlus, www.incontinentiapigmenti.it) and came from several different regions in Italy.

The inclusion criteria adopted to recruit the IP patients in this study were:

1. genetic homogeneity of the ip *locus*: all patients carried the *IKBKGdel* allele in the *IKBKG/NEMO* gene as a cause of IP;
2. familial data: none of the patients had any previous familial history of mental handicap;
3. italian mother tongue: all patients, recruited thanks to the collaboration of the Italian IP association (I.P.A.SS.I. Onlus), were tested using Italian language tests.

Age and education ranged respectively from 21–59 and 8–18 years (mean age = 33.3±13.02 years and mean education = 12.8±3.85 years). The study was approved by the local Ethics Committee of the IRCCS Santa Lucia Foundation in Rome, Italy. After a full explanation, participants or their parents gave their written informed consent according to the standards set by the local Ethics Commitee. All participants filled in the clinical questionnaire (approved by the International IP Consortium, www.ipif.org), which investigates all aspects of IP phenotype: skin, hair, teeth, nail, eye, and CNS defects.

### Neuropsychological analysis

To obtain a measure of the participants' general verbal and non verbal intelligence, we submitted them to a cognitive assessment using the Wechsler Adult Intelligence Scale-Revised (WAIS-R; Italian Version [21]). According to their educational level, participants were also submitted to a battery of tests examining reading [22], writing [23] and arithmetic [24] skills. The aim was to determine whether the participants with IP whose general intelligence was in the normal range had any specific *learning disabilities*. All participants were assessed individually in a dedicated room remote from noise or any other cause of disturbance. Reading competence was assessed with a standard, widely used reading achievement test for junior high school students (MT Reading Test [22]). In this test, two meaningful text passages were presented. The participants had to read the first passage aloud (within a 4-minute time limit). Speed (time in seconds per syllable) and accuracy (number of errors, adjusted for the amount of text read) were considered. To measure comprehension, participants had to read the second passage without a time limit and then respond to 15 multiple-choice questions with four alternatives. Raw scores were converted to z scores according to standard reference data [25]. To assess writing skills and orthographic accuracy, we administered the handwriting task, which consisted of writing a text read aloud by the examiner (battery for evaluating orthographic abilities in public schools [23]). To assess arithmetic competence, we used standardized arithmetic batteries (AC-MT 11–14; [26] MT-advanced 2 [27]) that included written (magnitude comparison tasks, number ordering tasks and logical-arithmetic problem solving) and oral calculations (mental calculation and general speed). The correct responses in each area were summed to obtain a total score. Stimulus materials and related reference norms varied depending on the grade level. Participants' performance was not invalidated by ocular defects because they wore glasses and their vision was normal or corrected to normal.

### Molecular analysis

DNA samples from all participants enrolled in this study were investigated by long-range PCR to reveal the presence of the *IKBKG/NEMO* gene deletion (*IKBKGdel*). This was performed with the EXPAND Long Template PCR system (Roche Mannheim, Germany) according to the manufacturer's protocol. Bardaro and colleagues [28] reported the specific primers to be used for the long-range PCR.

### Statistical analysis

All statistical analyses were performed with SPSS Statistics 19. Considering the small size of our sample, we investigated differences in verbal and performance IQ by the preliminary application of a non-parametric test (Wilcoxon test). However, as non-parametric statistics are less powerful than their parametric counterparts (and for us it was very important to detect even small effects), we also performed a parametric test (paired t-test). The alpha level was set at .05. We performed a Pearson correlation among VIQ, PIQ and FSIQ and brain disease with the alpha set at .05. To determine whether performance on the reading, writing and mathematics tests was correlated with the FSIQ, VIQ and PIQ scales and with the number of IP symptoms reported by the participants and assessed by the questionnaire, we performed Pearson correlations by setting the alpha level at .05.

## Results

Results of the clinical questionnaire revealed that, in addition to the presence of the typical IP skin defects, all IP patients enrolled in the study had one additional ocular, dental, hair or nail defect: 2 out of 10 had brain malformations; 2 out of 10 suffered from seizures; none had hemiparesis; and 6 out of 10 showed other symptoms (see Table 1).

**Table 1:** Clinical manifestations in 10 female patients with IP: Clinical score analysis.

Participants	Molecular defect of *NEMO/IKBKG*	Skin Defects	Ocular/Dental/Hair/Nail Defects	CNS Defects	Other Defects	Total Score Symptoms
Pt1	*exon4-10del*	2	3	0	0	5
Pt2	*exon4-10del*	2	3	0	1	6
Pt3	*exon4-10del*	4	4	0	0	8
Pt4	*exon4-10del*	2	4	0	0	6
Pt5	*exon4-10del*	1	3	0	0	4
Pt6	*exon4-10del*	2	2	0	1	5
Pt7	*exon4-10del*	5	3	0	2	10
Pt8	*exon4-10del*	3	1	0	1	5
Pt9	*exon4-10del*	2	2	2	1	7
Pt10	*exon4-10del*	3	4	3	1	11

The score represents the number of clinical manifestations in each organ system.

Skin defects include: vesicles, pustules, hyperkeratotic lesions, pigmented spots, and hypopigmentation.

Ocular defects include: refractive errors; nystagmus, strabismus, optic atrophy, and iris pigmentary abnormalities.

Dental defects include: partial anodontia, dental dystrophy, dental pulp defects, cone/peg shaped teeth, malocclusion, and gingival defects.

Hair defects include: hair atrophy, alopecia, and wooly hair naevus.

Nail defects include: ungual dystrophy.

CNS (central nervous system) defects include: ischemia, seizures, and cerebral atrophy.

Other defects include: congenital clubfoot, frequent infections, vascular diseases, cranial asymmetry, and breast atrophy.

The molecular test for IP not only confirmed the clinical diagnosis but also showed that the IP *locus* alteration was homogeneous in all patients. Indeed, all participants carried the exon4-10del *(IKBKGdel* allele, see inclusion criteria) as the cause of IP.

All participants were able to perform both the Verbal and Performance Scale of the WAIS-R. Seven women with IP (70%) had a full-scale IQ above 70 (FSIQ range 92–118) and three women with IP (30%) had intellectual deficiencies (FSIQ range 45–70). The mean scores were 92±25.16 for Full Scale IQ (FSIQ), 90.6±22.84 for Verbal IQ (VIQ), and 93.8.8±25.45 for Performance IQ (PIQ). The PIQ and VIQ were not significantly different in either the non-parametric (z = −1.25; p = 0.21) or parametric statistics (t_1,9_ = −1.29; p = 0.23), thus confirming the robustness of the present result. See Table 2 for details.

**Table 2:** IP individuals' WAIS-R IQs and raw scores.

Patients	Age (yrs)	Education (yrs)	IQ	Information	Digit Span	Vocabulary	Arithmetic	Comprehension	Similarities	VIQ	Picture Completion	Picture Arrangement	Block Design	Object Assembly	Digit Symbol	PIQ
Pt1	23	16	107	7	11	13	8	11	10	102	11	12	11	10	12	110
Pt2	49	8	96	5	5	8	8	10	8	91	9	10	16	7	5	105
Pt3	21	13	99	7	9	6	12	12	13	101	10	7	9	8	13	96
Pt4	33	18	106	8	12	13	12	12	12	109	12	8	12	10	10	103
Pt5	28	18	111	10	8	11	8	15	15	106	16	11	14	7	13	111
Pt6	59	13	110	10	5	10	8	16	9	107	8	7	5	10	9	112
Pt7	21	13	118	9	8	11	11	15	13	111	10	14	12	14	13	121
Pt8	44	8	58	4	2	6	3	4	7	66	7	1	1	2	2	57
Pt9	27	13	70	5	4	5	8	6	6	68	9	6	7	6	7	78
Pt10	28	8	45	1	1	1	1	1	1	45	2	3	1	1	2	45

**Table reports patients' IQ and subset scores on the WAIS-R.**

A Pearson correlation, which was carried out for VIQ, PIQ, FSIQ and the number of brain symptoms (malformations, seizures and hemiparesis) shown by all patients, revealed a significant negative correlation (VIQ: r = −0.8; p<.01; PIQ  =  r = −.72; p<.05; FSIQ  =  r = −.75; p<.05).

Five participants with an IQ above 70 who were submitted to tests evaluating arithmetic skills performed deficiently (83.33%; 5 out of 6); three participants submitted to tests evaluating reading skills performed deficiently (42.86%; 3 out of 7); and three participants had borderline performances (42.86%; 3 out of 7). None of the seven participants performed deficiently on the tests evaluating writing skills (see Tables 2, 3 for details).

**Table 3:** Performance scores of seven educated IP participants with IQs above 70 on reading, writing and mathematics tests.

Patient	Age (years)	FSIQ	Dictation	Reading (accuracy)	Reading (speed)	Reading (comprehension)	Arithmetic	Algebra	Geometry	Arithmetic Problems	Total Math Test	Mental Math (accuracy)	Mental Math (speed)	Arithmetic facts
Pt1	49	96	np	**−1.40** *	**−**0.05	−0.87#	np	np	np	np	np	np	np	np
Pt2	21	99	0.25	**−2.68** *	0.5	0.27	−0.76#	**−1.65** *	−0.53#	**−1.63** *	**−1.97** *	0.76	**−0.84** *	−1.03#
Pt3	33	106	0.96	0.40	−0.43	0.77	0.77	0.18	0.72	4.81	1.07	1.86	0.72	0.63
Pt4	28	111	1.32	−0.51#	0.4	0.27	0.26	0.18	1.35	**−2.44** *	−0.51#	1.86	−0.31#	0.42
Pt5	59	110	1.32	0.51	−0.02	0.77	**−2.3** *	**−2.11** *	**−1.8** *	2.39	**−2.1** *	1.31	0#	0.22
Pt6	21	118	0.96	**−2.56** *	**−1.67** *	−0.23 #	**−1.8** *	0.64	**−2.4** *	**−1.63** *	**−1.92** *	0.76	0.61	0.22
Pt7	23	107	0.6	−0.85#	−0.47	1.27	**−2.29** *	−0.27#	**−1.79** *	−0.83#	**−2.10** *	0.20	−0.59#	−1.45#

*pathological performance; # borderline performance; np  =  test not performed.

Scores for learning abilities are z-scores; pathological performances are defined in accordance with the normative data of the tests.

Pearson correlations were also calculated to determine whether performances on the reading, writing and mathematics tests were correlated with the FSIQ, VIQ and PIQ scales and with the number of IP symptoms self-reported by the participants, as assessed by the questionnaire. The analysis did not show any significant correlations between the number of IP symptoms and performance on tests assessing learning skills (see Table 4 for details).

**Table 4:** Correlation between WAIS-R scales, learning tests and number of IP symptoms.

	FSIQ	VIQ	PIQ	Dictation	Reading -accuracy	Reading-speed	Reading -comprehension	Arithmetic	Algebra	Geometry	Arithmetical Problems	Total Math Test	Mental math -accuracy	Mental math-speed	Arithmetic facts	Number of IP symptoms
FSIQ	1	.857*	.855*	.665	.100	−.628	.258	−.264	.543	−.343	−.148	−.086	.079	.606	.515	−.081
VIQ	.857*	1	.489	.657	.222	−.455	.470	.159	.505	−.065	.362	.388	.467	.952**	.848*	.028
PIQ	.855*	.489	1	.596	.031	−.717	−.050	−.458	.449	−.489	−.211	−.264	−.103	.468	.333	−.211
Dictation	.665	.657	.596	1	.644	−.089	−.082	.101	.171	.190	.248	.288	.661	.508	.797	−.438
Reading (accuracy)	.100	.222	.031	.644	1	.224	.520	.192	−.123	.339	.716	.494	.581	.282	.442	.149
Reading (speed)	−.628	−.455	−.717	−.089	.224	1	.091	.339	−.587	.603	−.054	.091	.316	−.682	−.173	−.054
Reading (comprehension)	.258	.470	−.050	−.082	.520	.091	1	−.183	−.274	.002	.459	.069	−.158	−.258	−.392	.629
Arithmetic	−.264	.159	−.458	.101	.192	.339	−.183	1	.364	.925**	.229	.889*	.765	.230	.495	.125
Algebra	.543	.505	.449	.171	−.123	−.587	−.274	.364	1	.202	−.152	.462	.132	.511	.306	.363
Geometry	−.343	−.065	−.489	.190	.339	.603	.002	.925**	.202	1	.124	.783	.771	−.037	.384	.152
Arithmetical Problems	−.148	.362	−.211	.248	.716	−.054	.459	.229	−.152	.124	1	.559	.434	.567	.423	.091
Total Math Test	−.086	.388	−.264	.288	.494	.091	.069	.889*	.462	.783	.559	1	.784	.521	.616	.259
Mental math (accuracy)	.079	.467	−.103	.661	.581	.316	−.158	.765	.132	.771	.434	.784	1	.439	.855*	−.308
Mental math (speed)	.606	.952**	.468	.508	.282	−.682	−.258	.230	.511	−.037	.567	.521	.439	1	.777	−.193
Arithmetic facts	.515	.848*	.333	.797	.442	−.173	−.392	.495	.306	.384	.423	.616	.855*	.777	1	−.507
Number of IP symptoms	−.081	.028	−.211	−.438	.149	−.054	.629	.125	.363	.152	.091	.259	−.308	−.193	−.507	1

The numbers in the table indicate Pearson's correlation coefficients (r).

*p<0.05;

**p<0.01 FSIQ: Full scale IQ; VIQ: Verbal IQ; PIQ: Performance IQ.

The VIQ was significantly correlated with the *Mental Maths Speed Test* (r = 0.95, p<0.01) and the *Arithmetic Facts Test* (r = 0.85, p<0.05). No other significant correlations were detected between the WAIS-R scales and the Learning tests.

## Discussion

Until now, studies on IP have mainly focused on the clinical symptoms that are always present in the IP phenotype or the neurological manifestations that require a significant medical intervention. In fact, the cognitive phenotype of IP has never been investigated. To our knowledge, this is the first study that has investigated the neuropsychological profile of IP patients by also assessing learning abilities in individuals with IP without intellectual disabilities. In contrast to what has been observed in individuals affected by other genetic syndromes (e.g., Cri-du-Chat Syndrome and Williams Syndrome) [29], [30], in IP patients we observed no discrepancy between verbal (VIQ) and performance (PIQ) IQ scores. In fact, our participants showed a homogeneous profile. Our sample was not wide, but it showed the same distribution percentage of neurological manifestations reported in the literature [11], [31].

We found that 2 patients out of 10 were affected by mental delays resulting from neurological signs and 1 patient out of 10 was affected by mental delay without any neurological signs. This latter observation is particularly interesting because it suggested that patients with IP might be affected by mental delay also when no neurological damage was present and allowed us to separate mental delay from the neurological framework. The remaining patients with IP manifested no mental retardation, but a detailed cognitive assessment allowed us to detect the presence of learning disabilities. In particular, the learning abilities most affected were arithmetic reasoning and reading skills. In the psychological literature, a comorbidity between dyslexia and dyscalculia is often reported; it is often associated with two largely independent cognitive deficits, namely, a phonological deficit in the case of dyslexia and a deficit in the number module in the case of dyscalculia [32]. In a review study, Jordan [33] reported that reading difficulties aggravated rather than caused mathematical difficulties because compensatory mechanisms associated with reading are less available when dyslexia and dyscalculia co-occur. Furthermore, in our sample reading and arithmetic difficulties co-occurred in 4 patients out of 6 (one participant refused to perform the arithmetic tasks). Reading seemed more affected in terms of accuracy than speed and comprehension, whereas all aspects of arithmetic were compromised (algebra, geometry, problem solving and arithmetic facts).

Our findings show that the IP group's performance was characterized by deficient arithmetic reasoning and reading skills. It is also possible that the heterogeneity of the cognitive phenotype is due to the *IKBKG/NEMO* mutation, which produces different phenotypic outcomes in mental functioning as well as physical characteristics.

Specifically, our results suggest that the *learning abilities* most affected are arithmetic reasoning and reading skills, which are reported here for the first time as specific deficits in IP. Therefore, when IP is diagnosed, patients should always be submitted to a cognitive assessment specifically focused on learning skills and should be included in preventive educational programs. The IP syndrome is very problematic because neither the type of *IKBKG/NEMO* genetic mutation nor the NEMO/IKKgamma protein affected domain is correlated with the severity of the IP phenotype [2]. Although the presence of *learning disabilities* also in IP patients without any mental retardation is a significant finding, the pathophysiology of this defect must still be addressed. One challenging approach would be to perform functional *MRI* (fMRI) studies of the brain to identify any impaired cerebral functions underlying learning deficits in this population.

In conclusion, the present study enrols learning disability amongst the CNS defects associated to IP disorder and supports a role of the IKBKG/NEMO gene as a genetic determinant of such a defect. The heterogeneity in the cognitive phenotype observed in our patient cohort could be a consequence of the *IKBKG/NEMO* mutation that might produce different phenotypic outcomes also in mental functioning. Accordingly, we would like to underline the importance of early assessment of learning abilities in individuals with IP who have no mental retardation to prevent the onset of deficits. A very recent study by Ginieri-Coccossis *et al.* [34] investigated the quality of life of children affected by specific *learning disabilities* and found that they had poorer emotional well-being, lower self-esteem and a higher level of dissatisfaction in their relationships with family and friends than children without these deficits. In line with this result, Michopoulou *et al.* [35] also observed psychological problems, such as anxiety, depression, anger and disruptive behaviour, in these children. To avoid stigmatizing effects due to ongoing difficulty in school performance, it is important to submit children with IP to precocious, targeted treatment. Compared with mental delay or other mental disabilities, *learning disabilities* do not seem to cause any major psychosocial impairment [36]. Nevertheless, failure to achieve academically can become a source of distress or external pressure for children and parents. If these difficulties are not treated at an early age, they can become long-standing and affect psychological development as well as the possibility of achieving professional fulfilment. Therefore, we would like to highlight the urgency of making an early diagnosis of learning disability in girls with IP by means of a comprehensive and thorough neuropsychological assessment, performed by a qualified clinician with regular follow-ups. Finally, we also suggest that re-educational training should be aimed towards developing appropriate skills and coping mechanisms.
